# Successive extraction of As(V), Cu(II) and P(V) ions from water using spent coffee powder as renewable bioadsorbents

**DOI:** 10.1038/srep42881

**Published:** 2017-02-21

**Authors:** Linlin Hao, Peng Wang, Suresh Valiyaveettil

**Affiliations:** 1 Department of chemistry, National University of Singapore, 3 Science Drive 3, 117543, Singapore; 2 State Key Laboratory of Urban Water Resource and Environment, School of Municipal and Environmental Engineering, Harbin Institute of Technology, Harbin, 150090, P. R. China

## Abstract

For the first time, renewable and easy accessible pre-bleached spent coffee powder coated with polyethylenimine (PEI) and ferric ions (Coffee-PEI-Fe) was used for the successive adsorption of As(V), Cu(II) and P(V) ions from spiked water samples. Fully characterized coffee-PEI-Fe was employed for batch mode experiments. Kinetic regression analysis showed that the adsorption processes of As(V) and P(V) anions follows a pseudo-second-order model, while the adsorption of Cu(II) ions fit with a pseudo-first-order model. The maximum adsorption capacities estimated by Langmuir model for As(V), Cu(II) and P(V) ions were 83.3, 200.1, and 50.2 mg/g, respectively. The simulated results revealed that the internal diffusion is the rate-determining step for the adsorptions of As(V) and Cu(II) ions, while film diffusion is the mass transfer resistance for the adsorption of P(V) ions on the surface of coffee-PEI-Fe. The successive adsorptions of adsorbates were achieved through electrostatic attraction between adsorbent surface and adsorbates. The dynamic column adsorption behavior of the adsorbent was described by Thomas model, which showed a good agreement with the experimental values (*q*_exp_). The results presented in this paper could be used for developing efficient adsorbent from renewable materials for water purification.

Large quantities of coffee beans are processed every year to quench the thirst for coffee drinkers around the world. However, about 45–50% of the total coffee beans (i.e. a few million tons) are disposed every year as spent coffee grounds^1^. In recent years, renewable resources have been explored as potential adsorbents for the extraction of different contaminants from water^2^. Spent coffee grounds comprises of lignin, hemicelluloses, cellulose, pectin and small quantity of extractives^3^. The abundant functional groups (such as hydroxyl group, amino group, and carboxyl group) on the surface of spent coffee grounds makes it a potential low-cost renewable adsorbent for pollutant removal from water^4^. In this study, readily available spent coffee grounds were pretreated with bleaching solutions to extract the cellulose nanofibers as low-cost adsorbents for the removal of arsenate, copper and phosphate ions from aqueous solutions. Pretreatment with bleaching solution is a convenient method, which ensures the removal of hemicelluloses, lignin and other soluble compounds from spent coffee to obtain cellulosic fibers as adsorbent^5^. Cellulose nanofibers are interesting adsorbents owing to their high specific surface area, large number of hydroxyl groups on the surface and exceptional mechanical properties such as low density, renewability, low cost, and low thermal expansion^6^.

Arsenic (As) pollution in water is a worldwide problem and has been considered as one of the major pollutants in potable water^7^. The occurrences of arsenic at elevated concentrations in groundwater has been well documented in many countries such as Argentina, Bangladesh, China, India, Mexico and Vietnam^8^. Fe-based sorbents have been extensively developed and showed good extraction efficiency for arsenic removal from contaminated water^9^. The high concentration of heavy metals such as copper and lead in water cause health hazards to living organisms^10^,^11^. Similarly, presence of phosphate in large quantities is known to deteriorate natural ecosystems, water quality and responsible for eutrophication problem of surface water^12^. Among the techniques currently available for pollutants removal from water, the adsorption process using biomass derived adsorbents is considered as one of the most promising techniques owing to high efficiency, easy to operate, economic and environmental factors^13^.

In the current work, pretreated spent coffee powder is used as a renewable adsorbent and a new protocol of layer-by-layer adsorption is developed to enhance the efficiency of adsorption. The pretreated coffee powder was treated with PEI, cross-linked to reduce water solubility and adsorbed with Fe (III) cations for enhancing the affinity towards arsenic anions in water. We hypothesized that adsorption of arsenic ions on the surface of the adsorbent lead to the development of a net negative surface charge, which could be used for the extraction of cationic pollutants such as Cu(II) ions from water and such alternating adsorption process can continue for a few adsorbates with opposite charges, before regenerating the adsorbent. Such successive extractions could be used in industrial cities where the effluents from each industry varies in chemical nature and composition. As a proof of concept, the pretreated coffee based adsorbent was used for the extraction of arsenic anions, copper cations and phosphate anions, successively (Fig. 1). Full characterization of the adsorbent and analytical data are given in the paper to demonstrate the efficiency of the pretreated spent coffee powder as potential adsorbent for water purification. To the best of our knowledge, this kind of layer-by-layer adsorption was seldom applied for water purification. In this study, successive adsorption based purification of real water sample was not tested, because we need to understand the interactions and interference of different cations and anions towards the adsorption efficiency. There is no need to discard or regenerate the exhausted adsorbent (e.g. adsorbent with As(V) on the surface), but can be reused as such for the adsorption of oppositely charged pollutants from water. Therefore, this layer-by-layer adsorption of alternatively charged pollutants from water may offer potential for reducing the overall cost of water purification process.

## Results and Discussion

### Characterization of Coffee Cell-PEI

Pretreated and purified spent coffee powder was fully characterized using a range of techniques, before employing for extraction studies.

#### Analysis of FT-IR spectra of adsorbents

**Figure 2:**
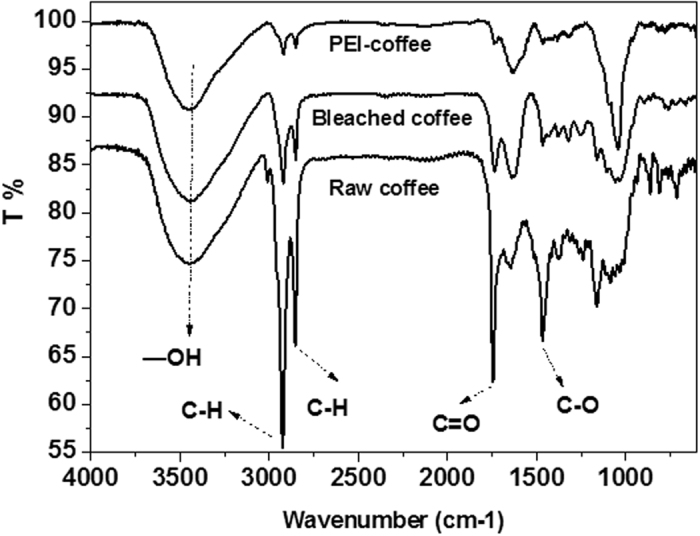
FTIR spectra of coffee powder before and after modification.

The changes in functional groups on the spent coffee powder before and after modification can be seen from FTIR spectra (Fig. 2). The broad absorption band at 3200–3600 cm^−1^ is characteristic of the stretching vibration of hydroxyl groups (from alcohols, phenols or carboxylic acid) and water adsorbed on the cellulosic fiber surface. The sharp peak at 2920 cm^−1^ was assigned to C-H asymmetric and symmetric stretch of the methylene groups (-CH_2_-) and methyl groups (-CH_3_)^14^,^15^. The band at 2853 cm^−1^ indicated aliphatic C-H stretching from the cellulose backbone^14^. The strong band at 1450 cm^−1^ of raw coffee grounds was assigned to -CH_2_ and -CH_3_ bending vibrations and/or the O-H bending peak due to the existence of phenols on the raw spent coffee powder surface^15^,^16^. The wide peak at 1100–1300 cm^−1^ may be assigned to the C-C vibration of the cellulose^17^. After PEI modification, all such bands were weakened and a new peak appeared at 1053 cm^−1^ is assigned to the C-N stretching vibration^18^.

#### Scanning electron microscopy and elemental analysis

**Figure 3:**
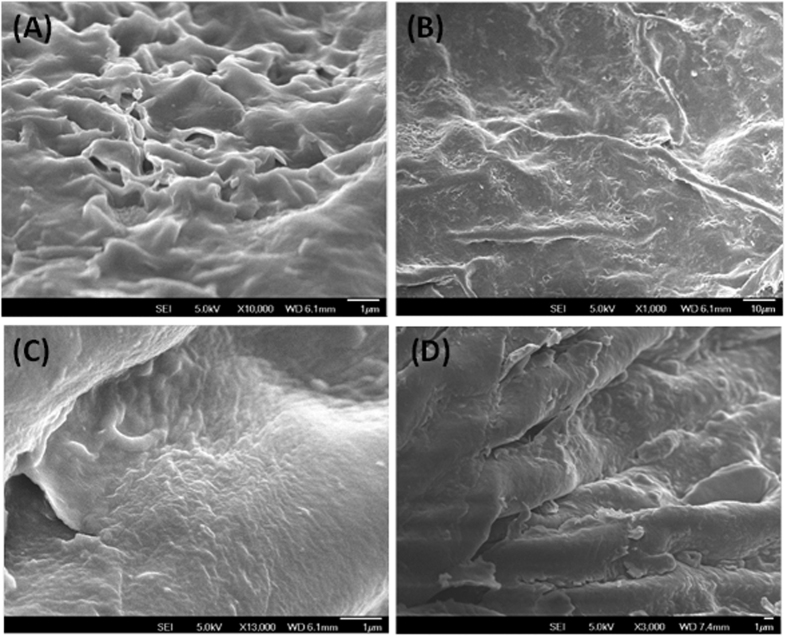
SEM images of raw coffee powder (**A**), bleached coffee powder (**B**), coffee-PEI (**C**) and after successive adsorption of pollutants, coffee-PEI-Fe-As-Cu-P (**D**).

Figure 3 depicts the SEM images of the raw coffee powder (Fig. 3A), pretreated spent coffee cellulose (Fig. 3B), Coffee-PEI (Fig. 3C) and Coffee-PEI-Fe after successive adsorption of As(V), Cu(II) and P(V) (Fig. 3D). The raw coffee powder surface is rough with a variety of flaky protuberances. After the bleaching pretreatment, a smooth surface was observed and the flaky protuberances disappeared. After coating with PEI and cross-linking with gluteraldehyde, the roughness of the adsorbent surface was reduced (Fig. 3C). The surface of Coffee-PEI-Fe was also rough and small lumps were observed after successive adsorption of As(V), Cu(II) and P(V) ions (Fig. 3D). As expected, the elemental analysis showed an increase in nitrogen content from 0.5% to 3% after coating the surface with PEI. No significant leaching of PEI from the adsorbent into water was observed during the washing or extraction experiments as nitrogen content in the aqueous fractions was non-detectable or insignificant. Elemental analysis of the coffee-PEI-Fe showed a Fe content of around 0.36%. After consecutive adsorption of As(V), Cu(II) and P(V) ions (using an initial concentration of each adsorbate solution of 5 mg/L), the elemental concentrations of As, Cu and P on the adsorbent surface were 0.14%, 0.76%, and 0.49%, respectively. Elemental analysis data of the adsorbents are given in Table 1.

### Batch adsorption of As(V), Cu(II) and P(V) ions using coffee-PEI-Fe adsorbent

#### As(V) anion adsorption on coffee-PEI-Fe adsorbent

**Figure 4:**
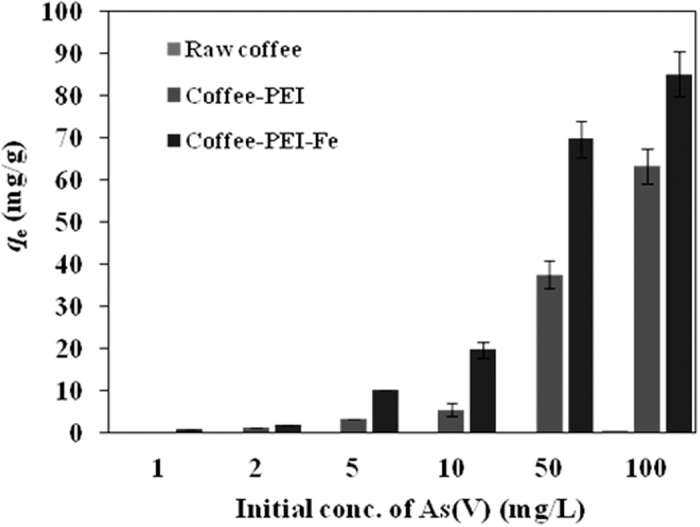
As(V) adsorption on raw coffee, coffee-PEI and coffee-PEI-Fe (The adsorbent concentration is 0.5 g/L, the initial pH is 7.1, at room temperature).

The comparison of As(V) adsorption on spent coffee powder, coffee-PEI and coffee-PEI-Fe are shown in Fig. 4. The initial concentration of As(V) anion used was 1, 2, 5, 10, 50, 100 mg/L and the raw spent coffee showed a maximum adsorption capacity of 0.2 mg/g for As(V) ions. However, the adsorption ability was significantly enhanced after treatment with PEI, followed by iron loading processes. The adsorption capacity of As(V) ions on coffee-PEI and coffee-PEI-Fe is increased significantly with an increase of initial concentrations. The maximum adsorption capacity of coffee-PEI was 63.1 mg/g while that of coffee-PEI-Fe was 85.1 mg/g at the initial As(V) ion concentration of 100 mg/L. The protonated amine groups of PEI were responsible for the increased As(V) anion extraction by coffee-PEI *via* electrostatic attraction. Similarly, the loading of Fe(III) cations on coffee-PEI-Fe surface significantly enhanced As(V) anion adsorption to 85.1 mg/g through the formation of Fe-As complex^19^. A comparison of three adsorbents, raw coffee, coffee-PEI and coffee-PEI-Fe, demonstrated that the incorporation of PEI and Fe on the surface contributed to the enhancement of As(V) anion extraction by the pretreated spent coffee powder (Fig. 4). A comparison of adsorbents reported in the literature is given in the Supplementary Information (Table S1).

#### Successive extraction of As(V), Cu(II) and P(V) ions on coffee-PEI-Fe adsorbent

**Figure 5:**
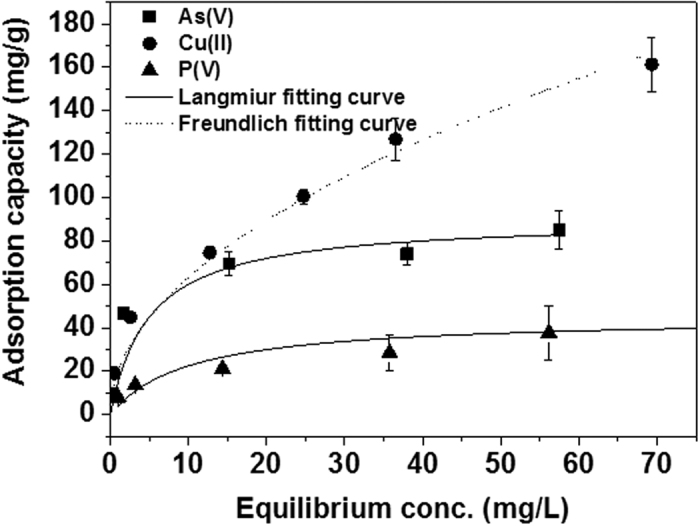
Langmuir and Freundlich adsorption isotherms for As(V), Cu(II) and P(V) ion adsorption of on coffee-PEI-Fe adsorbent. Experiments were done using the initial concentrations of As(V), Cu(II) and P(V) ions varied from 1 to 150 mg/L, the adsorbent concentration at 0.5 g/L, the initial pH of 7.1, at room temperature.

Figure 5 shows the successive extraction of As(V), Cu(II) and P(V) ions on coffee-FEI-Fe adsorbent. It can be seen that As(V) ion extraction on coffee-PEI-Fe increased from 1 to 85 mg/g with an increase in initial concentration of arsenate from 5 to 100 mg/L. Meanwhile, Cu(II) ion extraction capacity was also increased with an increasing initial concentrations, and the maximum extraction capacity was reached to 161 mg/g. After As(V) anion adsorption, the effective negative charges on the surface of coffee-PEI-Fe favors the adsorption of Cu(II) cations from solution. Similarly, adsorption of Cu(II) cations enhances the net positive charges on the surface and facilitate the adsorption of P(V) anions with an adsorption capacity of about 48 mg/g, when the initial concentration of P(V) anion used was 100 mg/L. Thus, layer-by-layer adsorption of oppositely charged ions was achieved through the maximization of electrostatic attraction between the ions. We hope that such successive removal of pollutant will be useful for treating effluents from various industries, in which the chemical nature of the pollutants is well established.

In order to estimate the change of surface charge during the consecutive adsorption, the zeta potentials of all adsorbents such as raw coffee cellulose, coffee-PEI, coffee-PEI-Fe, after As(V) anion adsorption (coffee-PEI-Fe-As), after Cu(II) cation adsorption (coffee-PEI-Fe-As-Cu) and after adsorption of P(V) anions (coffee-PEI-Fe-As-Cu-P), were measured and summarized in Table 2. The raw coffee cellulose after bleaching showed a zeta potential of −29.8 mV, indicating a negatively charged surface. After modification with PEI, the negative zeta potential was decreased to −3.92 mV owing to the presence of protonated amine groups on the surface. The zeta potential was increased to +41.6 mV after adsorption of Fe (III) ions on the surface, which facilitates the adsorption of negatively charged arsenate ions from solution. As expected, the adsorption of As(V) ions resulted in the reduction of positive zeta potential to +1.1 mV, which was again increased to +41 mV after Cu(II) cation adsorption. Similarly, adsorption of P(V) anion on the surface reduced the zeta potential to +21.1 mV. The consecutive increase and decrease in Zeta potentials indicated the layer-by-layer adsorption of cations and anions from solution.

Langmuir and Freundlich isotherm models were used to describe the adsorption isotherms as shown in Fig. 5, the fitting parameters were summarized in Table 3. For As(V) and P(V) adsorption, the Langmuir model showed a better fit with the adsorption isotherms (R^2^ > 0.99) than Freundlich model (R^2^ between 0.97~0.98). For Cu(II), the Freundlich model fitted much better than Langmuir model. The maximum adsorption capacities of As(V), Cu(II) and P(V) ions estimated by Langmuir model are 83.3, 50.2 and 200.1 mg/g, respectively. A comparison of Langmuir constant^19^ indicated that the adsorption affinity towards Cu(II) was stronger than As(V) and P(V) ions, owing to the presence of amine functional groups on the surface which enhances the affinity towards Cu(II) ions.

#### Adsorption kinetics

**Figure 6:**
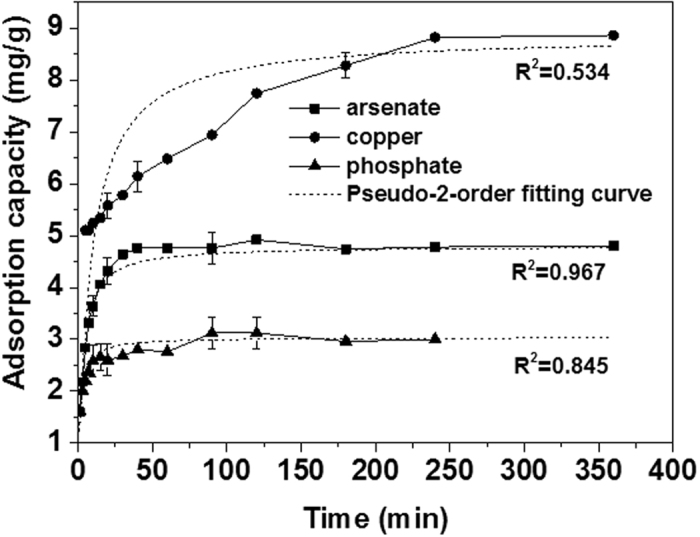
Kinetic curves of As(V), Cu(II) and P(V) adsorption on coffee-PEI-Fe in deionized water (Initial concentration for each element is 5 mg/L, respectively, adsorbent dose is 1 g/L, in room temperature).

For the adsorption of As(V) and P(V) anions on the adsorbent surface (as shown in Fig. 6), the initial adsorption rates within the first 25 min are relatively fast, followed by a slower reaction rate, and the adsorption equilibrium was achieved within 100 min. For Cu(II) ions, the adsorption rate is much faster owing to strong interaction between copper ions and amines on the surface, and the rate remain constant for the period of up to 250 min and then shows a plateau. It is conceivable that two different mechanisms may be involved for the adsorption of As(V), P(V) and Cu(II) on the surface of adsorbent. To understand the kinetics of arsenate adsorption onto coffee-PEI-Fe surfaces, the data were analyzed using both pseudo-first-order and pseudo-second-order kinetic models. The parameters of these two models were summarized in Table 4.

**Figure 7:**
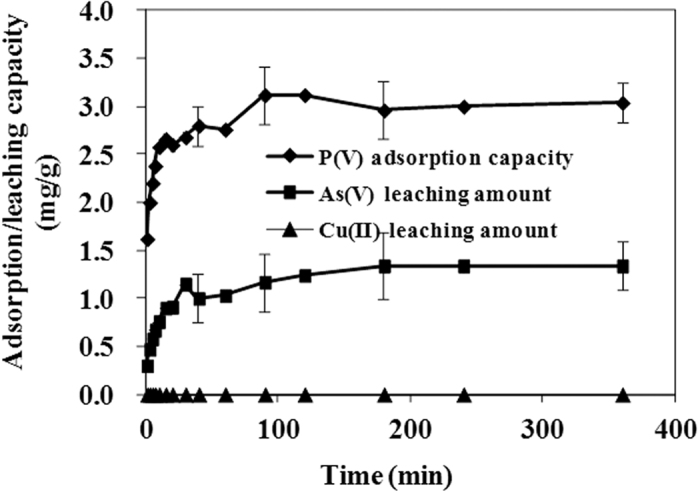
Arsenic and copper leaching during the adsorption of P(V) ions on coffee-PEI-Fe in deionized water (Initial concentration of P(V) is 5 mg/L, adsorbent dose is 1 g/L, at room temperature).

As can be seen from Table 4, the kinetics involved in the adsorption of As(V) and P(V) anions can be well described by pseudo-second-order model, implying a chemical adsorption with strong affinity to the surface. The correlation coefficient R^2^ is 0.992 and 0.845 for As(V) and P(V), respectively. The *k*_2_ value of 0.22 for P(V) ions is much higher than that of 0.07 for As(V) anions, indicating that coffee-PEI-Fe exhibited a stronger affinity towards P(V) than As(V) anions. Moreover, the observed leaching of adsorbed As(V) anion into the solution during P(V) adsorption could be due to a competitive adsorption between As(V) and P(V) anions from solution. As can be seen from Fig. 6, the adsorption curve of P(V) anions is similar in shape with that of the leaching curve of arsenic anions. At the same time, leaching of Fe(III) ions during the Cu(II) adsorption was not observed (Fig. 7). For the adsorption of Cu(II) cations, the kinetic data fitted much better to the pseudo-first-order model with a R^2^ value of 0.986 and strong electrostatic interaction between the adsorbent and adsorbate is the dominant adsorption mechanism. Moreover, Cu(II) ions form a blue color copper-amine complex with the grafted PEI molecule.

**Figure 8:**
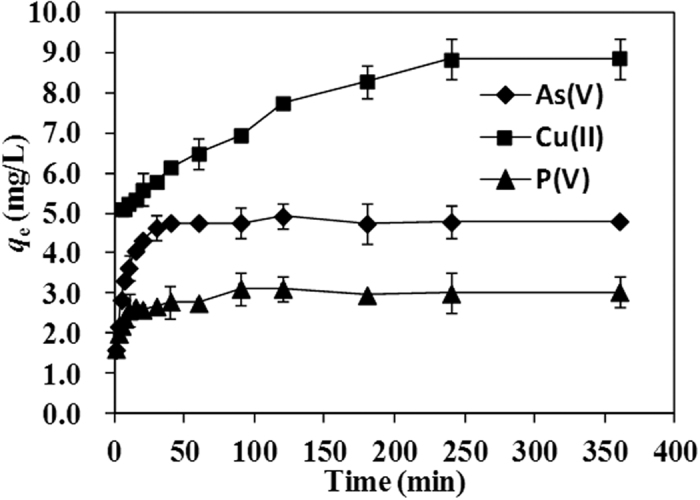
Kinetic curves of mixed As(V), Cu(II) and P(V)ion adsorption on coffee-PEI-Fe surface from deionized water. Initial concentration for each element is 5 mg/L, adsorbent dose was 1 g/L and at room temperature.

In order to further investigate the different affinity of coffee-PEI-Fe surface towards adsorption of As(V), Cu(II) and P(V) ions, a kinetic study was conducted with all three ions mixed together (the initial concentrations for the three ions were 5 mg/L). As can be seen from Fig. 8, the kinetic curves are similar to that given in Fig. 6, but the adsorption capacities for As(V), Cu(II) and P(V) ions are decreased, suggesting a competitive adsorption on the adsorbent surface. Some of the amine groups on the surface of Coffee-PEI-Fe powders can be neutral, while some are cationic in nature. The neutral electron rich amino groups bind to Cu(II) cations while the cationic groups interact strongly with As(V) and P(V) anions. Unlike systematic adsorption performed one ion after the other, the mixed ions may compete for the surface functional groups, which leads to a decrease in adsorption capacities for each ion.

#### Dynamic column study

**Figure 9:**
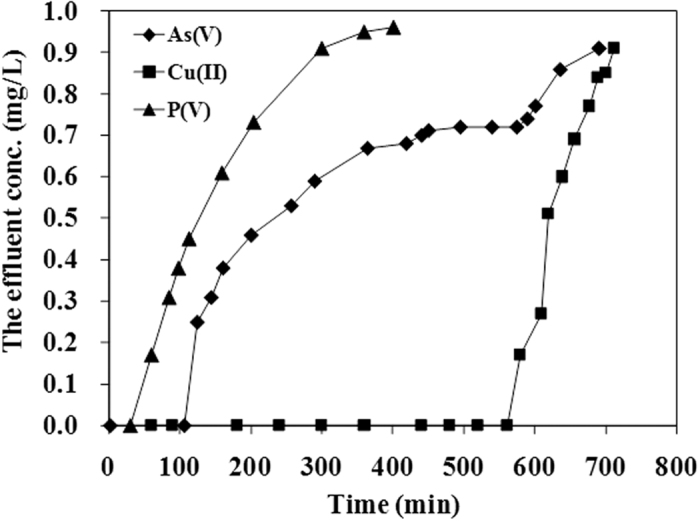
The breakthrough behavior of the column runs for adsorption of As(V), Cu(II) and P(V) ions on coffee-PEI-Fe adsorbent. Initial concentration of As(V), Cu(II) and P(V) ions is 1 mg/L; pH 7.0 ± 0.2, flow velocity is 10 mL/min.

Batch experiments were performed to evaluate the equilibrium capacity of adsorbent for adsorbate present in water. But in practical industrial water treatment processes, the experimental data obtained from the laboratory scale fixed bed column experiments are helpful for checking the potential applicability at the industrial scale. Column adsorption offers a more realistic simulation by replicating the batch treatment. Therefore, a dynamic column adsorption experiment was conducted to determine the mass transfer mechanism and various types of column parameters. As shown in Fig. 9, the duration to reach the breakthrough point for 1 mg/L for As(V) ions, needs 110 min at a flow velocity of 10 mL/min, while it needs 60 min to reach the breakthrough point for 1 mg/L for P(V) ions at the same flow velocity. For Cu(II) ions, it needs a much longer time of 540 min to reach the breakthrough point.

It is generally believed that the process of mass transfer is controlled by four independent processes^20^, which include bulk transport, film diffusion (mass transfer across the external boundary), intra-particle diffusion (diffusion within the pores of the adsorbent), and chemical reaction (adsorption at a special site on the surface). In order to further investigate the mechanism of mass transfer, the kinetic data was simulated using the models discussed in detail by *Fulazzaky*^21^. It is briefly introduced as follows:


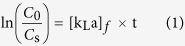


where *C*_*0*_ is the initial concentration of adsorbate (in mg/L), *C*_*s*_ is the concentration of adsorbate in the eluent at time *t* (in mg/L), [*k*_*L*_*a*]_*f*_ is the external mass transfer factor (or film mass transfer factor) (in 1/h), *t* is the reaction time (in h). Monitoring C_*s*_ at outlet of the column after the breakthrough point is important for modeling.


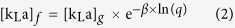


where [*k*_*L*_*a*]_*g*_ is the global mass transfer factor in 1/h.

By substituting Eq. (1) into Eq. (2) yields a continuous equation valid in determining the variation of mass transfer factor.


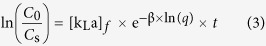


A deduction of Eq. (3) mathematically gives a linear expression below:


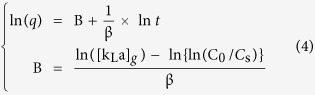


where *q* is the cumulative quantity of solute on adsorbent (in mg/g), *β* is the adsorbate-adsorbent affinity parameter (in g h/mg).


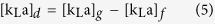


where [*k*_*L*_*a*]_*d*_ is the internal mass transfer factor (in 1/h).

**Figure 10:**
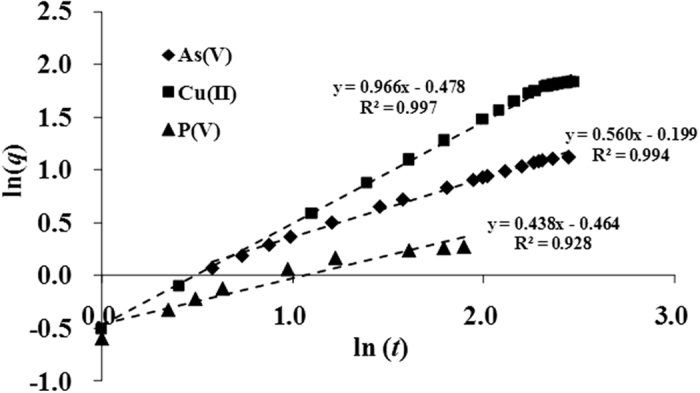
Linear regression analysis for the adsorption of As(V), Cu(II) and P(V) ion adsorption on coffee-PEI-Fe adsorbent.

A plot (Fig. 10) of ln(*q*) versus ln(*t*) gives a straight line with an intercept at *B* and 1/*β* as slope with the correlation coefficient of 0.928, indicating that the use of parameter B and *β* is reasonable for investigating mass transfer potential and adsorbate-adsorbent affinity for As(V), Cu(II) and P(V) ions on coffee-PEI-Fe adsorbent.

**Figure 11:**
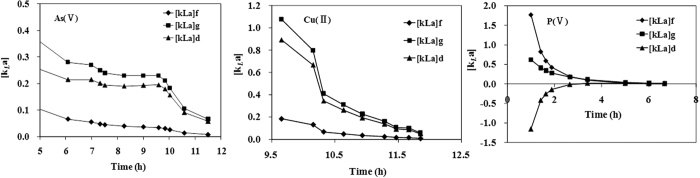
The variations of global, external and internal mass transfer factor pursuant to the running time for the adsorption of As(V), Cu(II) and P(V) ions on the adsorbents.

The variations of global, external and internal mass transfer factors are shown in Fig. 11. The curves of [k_*L*_a]_*g*_, [k_*L*_a]_*f*_, [k_*L*_a]_*d*_ for these three ions descended progressively with increase in running time and the values of both [k_L_a]_*f*_ and [k_L_a]_*d*_ approached to zero when the coffee-PEI-Fe surface was saturated through adsorption.

**Figure 12:**
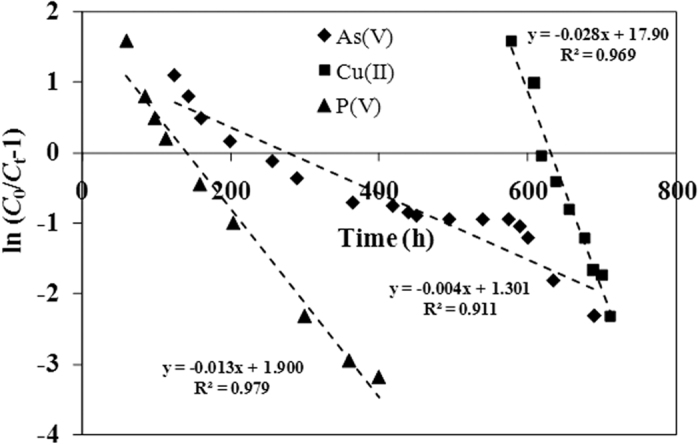
Linear Thomas model fit of breakthrough data for As(V), Cu(II) and P(V) ion adsorption on coffee-PEI-Fe surface.

For As(V) and Cu(II) ions, the internal mass transfer [*k*_*L*_*a*]_*d*_ constituted a larger proportion of the global mass transfer [*k*_*L*_*a*]_*g*_, indicating that the resistance of mass transfer is dependent on the internal mass transfer. The chemical interactions between As(V) anions and the Fe(III) cations on the surface are relatively important for enhancing the As(V) ion adsorption. Arsenic tends to form inner-sphere complex with iron oxyhydroxide through mechanism of ligand exchange. Such chemisorption is also indicated by the simulation with the pseudo-second-order model. Cu(II) ions form the expected copper-amine complex on the coffee-PEI-Fe surface. For P(V) anions, the external mass transfer [*k*_*L*_*a*]_*d*_ constituted a larger proportion of the global mass transfer [*k*_*L*_*a*]_*g*_, indicating the film transfer is the main resistance for P(V) anion adsorption on the surface of coffee-PEI-Fe adsorbent. Thomas model was used to fit the data from tests of column adsorption (R^2^ > 0.92) (Fig. 12, Table 5). The results of column adsorption capacity indicated a good agreement between the values (*q*_0_) generated using Thomas model and the experimental values (*q*_exp_).

#### Recycling of the adsorbents

**Figure 13:**
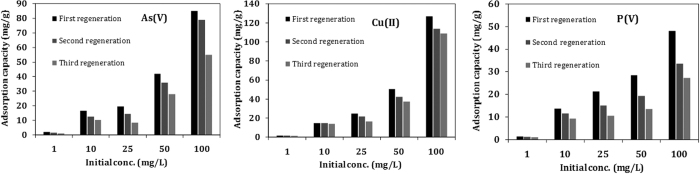
Regeneration of As(V), Cu(II) and P(V) adsorption on coffee-PEI-Fe surface.

In the practical application, the stability and reuse of the adsorbents are usually important factors because of the economic necessity. However, adsorbent based on biomass are readily available at a low cost and may not be recycled. Desorption of As(V), Cu(II) and P(V) from coffee-PEI-Fe surface were carried out by washing with 5% NaOH solution (100 mL) or 5% H_2_SO_4_ solution (100 mL), respectively. The desorption process occurred via ionization of surface functional groups and replacement of the adsorbed As(V) and P(V) anions by OH^−^, and replacement of Cu(II) ions by H^+^ ions. Data from desorption studies are shown in Fig. 13. The adsorption efficiency decreased with an increasing number of cycles, i.e., over 70% of efficiency for As(V) and Cu(II) and over 60% of efficiency for P(V) were obtained in the third adsorption-desorption cycles. This indicates that the adsorption of As(V), Cu(II) and P(V)on coffee-PEI-Fe was reversible and the adsorbent could be recycled after proper treatments.

## Conclusion

In summary, renewable low-cost spent coffee powder was used as an effective adsorbent for the removal of As(V), Cu(II) and P(V)ions from water. The raw spent coffee grounds were bleached thoroughly to remove lignin, hemicellulose and maximize the cellulose content. Iron was adsorbed on the PEI coated spent coffee cellulose surface to facilitate successive adsorption of As(V), Cu(II) and P(V) ions using the changes in net surface charges after adsorption of each ions. The maximum experimental adsorption capacities of coffee-PEI-Fe were 83.3, 200.1, and 50.2 mg/g for As(V), Cu(II) and P(V) ions, respectively. The kinetic study using the mixture of three ions in the same solution suggested coffee-PEI-Fe exhibited the strongest affinity towards Cu(II) ions through electrostatic attraction and metal coordination, followed by P(V) and As(V) anions. The rate-limiting step in the adsorption of As(V) and Cu(II) ions depends on the internal mass transfer resistance, while the film mass transfer mainly hinder the adsorption of P(V) ions on coffee-PEI-Fe surface. The column data showed a good fit with Thomas model and a good agreement between the simulated and experimental data. Our study concludes that the treated spent coffee ground is an interesting renewable adsorbent for the removal of various pollutants from contaminated water. Besides the As(V), Cu(II) and P(V) ions, coffee-PEI-Fe adsorbent can be used as a potential adsorbent for the removal of other dissolved pollutants.

## Methods

### Synthesis of modified coffee grounds adsorbents

The spent coffee materials were collected from local coffee shops and washed several times with water to remove soluble compounds. The pretreatment of the spent coffee (~1000 g) was conducted repeatedly using commercial bleach solution (5%, 200 mL) for several times until the color of the spent coffee powder changed from black to white. The floating cellulose fibers were separated (~100 g) and stirred with 10% polyethyleneimine (PEI, 100 mL) solution for 2 hrs, followed by crosslinking with aqueous solution of glutaraldehyde (0.5 mL, 25%). The product was washed with deionized water to remove excess reagents, dried in air and named as coffee-PEI. In order to increase the adsorption affinity towards arsenic anions, different batches of coffee-PEI (20 g) were stirred in aqueous Fe(NO_3_)_3_ solutions separately with different initial concentrations (100, 250, 500, 1000 mg/L) of Fe(NO_3_)_3_ solutions (250 mL) for 4 hr to adsorb of Fe (III) ions, filtered, washed with water and dried to obtain a series of coffee-PEI-Fe adsorbents. In order to establish the amount of iron loading, coffee-PEI-Fe (0.2 g) was digested with 50 ml of concentrated nitric acid and the solution was analyzed for iron concentration using elemental analysis.

### Successive adsorption of As(V), Cu(II) and P(V) on the adsorbent

#### Batch adsorption experiments

The initial concentrations of 1, 5, 10, 50, 75, 100 mg/L As(V) solutions were used for arsenic adsorption experiments using coffee-PEI-Fe (0.5 g) suspended in water (100 ml). The deionized water was used for making all solutions without adjusting the initial pH of 7.1. The adsorption capacity for arsenic was determined using the equation below:


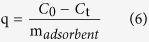


where *q* is the amount of As adsorbed per unit mass of the adsorbent (mg/g) at a given time *t*; *C*_*0*_ is the initial concentration of As (mg/L); *C*_*t*_ is the concentration of As (mg/L) at a given time *t*; *m*_*adsorbent*_ is the mass of the adsorbent (g/L).

After arsenic adsorption, the adsorbent (coffee-PEI-Fe-As) was filtered and collected for Cu(II) ion adsorption experiment. The Cu(NO_3_)_2_ solutions with initial concentrations of 1, 5, 10, 50, 75, 100 mg/L were used to study the adsorption of Cu(II) ions and extraction capacity was determined using the equation (6). After the extraction of Cu(II) ions, the adsorbent (designated as coffee-PEI-Fe-As-Cu) was filtered and collected for phosphate anion extraction. The NaH_2_PO_4_ solutions with concentrations of 1, 5, 10, 50, 75, 100 mg/L were used to study the adsorption capacities for P(V) ions. The final adsorbent was designated as coffee-PEI-Fe-As-Cu-P. The Langmuir and Freundlich models are frequently used to simulate the adsorptive isotherms data. The Langmuir equation (7) assumes that adsorbates form continuous monolayer on energetically equivalent sites on the adsorbent surface^22^.


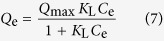


where *Q*_e_ (mg/g) is the equilibrium adsorption capacity, *Q*_max_ (mg/g) is the maximum adsorption capacity, *C*_e_ (mg/L) is the equilibrium arsenic concentration, *K*_L_ is a constant related to the binding energy. The separation factor *R*_*L*_, as a characteristic parameter of this isotherm, is expressed as:


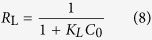


The Freundlich model is usually seen as an empirical equation (9), which is based on the assumption that multilayer adsorption on an energetically heterogeneous surface^23^.


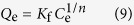


where *Q*_e_ (mg/g) is the amount of adsorbed arsenic per unit mass of the adsorbent at equilibrium, *C*_e_ (mg/L) is the equilibrium arsenic concentration, *K*_f_, *n* are parameters related to the adsorption capacity and the intensity of adsorption.

#### Kinetic studies

Coffee-PEI-Fe (1.0 g) was dispersed in As(V) solution (1000 ml, 5 mg/L) to determine adsorption capacity at different time intervals. The initial solution pH was around 7.0 and no changes were done during the adsorption experiments. The kinetics study was carried out using an orbital shaker operating at 250 rpm. Time point collections of samples (5 mL) were done at 5, 10, 15, 20, 30, 40, 60, 90, 120, 180, 240, 360, 1440 mins. Arsenic concentrations were detected by an Inductively Coupled Plasma- Optical Emission Spectroscopy (ICP-OES). The As-laden coffee-PEI-Fe adsorbent was collected by filtration for the kinetic experiment with Cu(II) ions. The initial concentration of Cu(NO_3_)_2_ solution was kept at 5 mg/L and the experimental procedure was repeated. After that, the Cu-As-laden coffee-PEI-Fe was collected by filtration and used for the extraction of P(V) ions from aqueous solution (1000 ml, 5 mg/L). In all cases, three consecutive runs were conducted and the solution samples and saturated adsorbents were collected for elemental analysis (EA). The average of the three values are reported as the final results.

The pseudo-first-order and pseudo-second-order kinetic models are commonly used to simulate As adsorption kinetics on various adsorbent surfaces^24^. The first pseudo-first order equation used is,


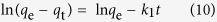


where *q*_t_ is the quantity of arsenic adsorbed at a time *t* (mg/g), *q*_e_ the quantity of arsenic adsorbed at equilibrium (mg/g), *k*_2_ (g· mg^−1^·min^−1^) is the rate constant.

The pseudo-second-order kinetic model^24^ is expressed as follows:


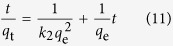



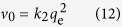


where *q*_t_ is the quantity of arsenic adsorbed at time *t* (mg/g), *q*_e_ the quantity of arsenic adsorbed at equilibrium (mg/g), *k*_2_ (g·mg^−1^·min^−1^) is the rate constant and *v*_0_ (mg·g^−1^·min^−1^) is the initial adsorption rate.

#### Column test

Dynamic adsorption experiments were conducted using a polyethylene column with10 cm in height and 2.0 cm inner diameter. Cotton was placed at the bottom of column to prevent discharge of adsorbents from the tubes. Coffee-PEI-Fe (1 g) was filled into the column (5 cm) and As(V) solution (1 mg/L) was added dropwise into the column. The flow velocity was kept at 10 mL/min and samples were collected at regular time intervals to determine the concentration of arsenic ions present in the filtered solution. After about 700 min run of As(V) adsorption, the feed solution was changed to Cu(II) (1 mg/L) and procedure was repeated. Effluent was collected at regular time intervals to determine the concentration of copper remained in solution. After about 1000 min run of Cu(II) adsorption, the feed solution of phosphate (1 mg/L) was added into the column, the procedure was repeated again and the effluents were collected for analyses.

Several models such as Thomas^20^, Yoon-Nelson^20^, Wolborska^21^ and Adams–Bohart models^25^ were used in the literature to simulate the column tests data. In this study, Thomas model was used to simulate the column test data, assuming the second order reversible reaction kinetics and the data follows the Langmuir isotherm model. The Thomas model was described as the following equation:


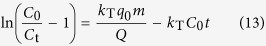


where, C_*0*_ and C_*t*_ are the influent and effluent concentrations (mg/L), *q*_0_ is the adsorption capacity (mg/g), *k*_*T*_ is the Thomas model constant (mL/mg · min), and *t* stands for total flow time (min). Q is the volumetric flow rate (mL/min). Values of *k*_T_ and *q*_0_ are determined from the linear plot of ln[(*C*_0_/*C*_t_) − 1] against *t*.

### Analytical determinations

FTIR spectra of the coffee cellulose before and after functionalization were recorded within the range of450~4000 cm^−1^ using a Bruker ALPHA FT-IR spectrophotometer using KBr as matrix. Scanning Electron Microscope (SEM, JEOL JSM-6701F field emission scanning electron microscope) was used to observe the changes in morphology of spent coffee powder before and after modification. Concentrations of As, Cu, P, and Fe ions in solution were determined using Inductively Coupled Plasma-Optical Emission Spectroscopy (Dual-view Optima5300 DV). Elemental analyses were done using an Elementar Vario Micro Cube instrument. Zeta potential measurements were done using Malvern Zetasizer Nano-ZS90 instrument.

## Additional Information

**How to cite this article**: Hao, L. *et al*. Successive extraction of As(V), Cu(II) and P(V) ions from water using spent coffee powder as renewable bioadsorbents. *Sci. Rep.* **7**, 42881; doi: 10.1038/srep42881 (2017).

**Publisher's note:** Springer Nature remains neutral with regard to jurisdictional claims in published maps and institutional affiliations.

## Supplementary Material

Supplementary Information

## Figures and Tables

**Figure 1:**
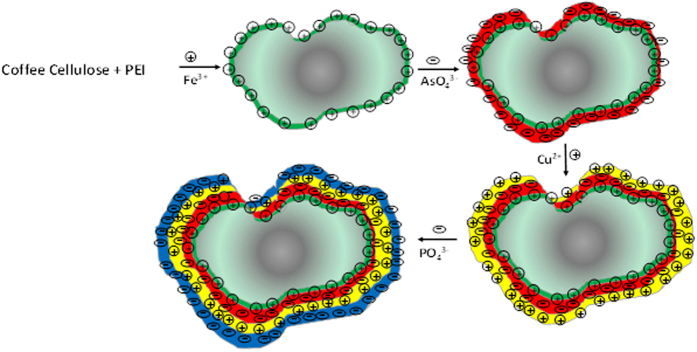
Cartoonistic representation of the successive adsorption of cations and anions form water solution on pretreated coffee powder surface.

**Table 1:** Elemental analysis of raw coffee powder, coffee-PEI-Fe and coffee-PEI-Fe-As-Cu-P (% based on dry weight).

Elements	Content (*Wt*%)		
	Raw coffee	Coffee-PEI-Fe	Coffee-PEI-Fe-As-Cu-P
C	42.5	44.3	43.9
H	6.2	6.4	7.1
N	<0.5	3.0	4.3
Fe	—	0.36	0.37
As	—	—	0.14
Cu	—	—	0.76
P	—	—	0.49

— Not Detected.

**Table 2:** The Zeta potentials of different materials prepared in this study.

Materials	Zeta potentials (mV)
Raw coffee cellulose	−29.8
Coffee-PEI	−3.92
Coffee-PEI-Fe	41.6
Coffee-PEI-Fe-As	1.1
Coffee-PEI-Fe-As-Cu	41
Coffee-PEI-Fe-As-Cu-P	21.2

**Table 3:** The parameters of isotherm models for As(V), Cu(II) and P(V) adsorption on coffee-PEI-Fe in deionized water at pH 7.1.

Models	*Langmuir*			*Freundlich*		
Species	*K*_*L*_(*L*/*mg*)	*Q*_*max*_(*mg*/*g*)	*R*^*2*^	*K*_*F*_(*mg*/*g*)(*L*/*mg*)^−*1*/*n*^	*n*	*R*^*2*^
As(V)	0.75	83.3	0.992	37.1	4.9	0.973
Cu(II)	0.09	200.1	0.949	22.7	2.1	0.975
P(V)	0.07	50.2	0.991	8.1	2.6	0.983

**Table 4:** The parameters of kinetic models for As(V), Cu(II) and P(V) ion adsorption on coffee-PEI-Fe.

Process	Pseudo-first-order model		Pseudo-second-order model		
	*k*_1_ (g·mg^−1^·min^−1^)	*R*^*2*^	*v*_0_ (mg·g^−1^·min^−1^)	*k*_2_ (g·mg^−1^·min^−1^)	*R*^*2*^
As(V)	0.06	0.813	1.61	0.07	0.967
Cu(II)	0.01	0.986	0.79	0.01	0.534
P(V)	0.03	0.683	2.03	0.22	0.845

**Table 5:** Thomas model constants for As(V), Cu(II) and P(V) adsorption on coffee-PEI-Fe.

Elements	Z (cm)	*v* (mL/min)	C_0_ (mg/L)	*k*_T_ (mL/mg · min)	*q*_0_ (mg/g)	*q*_exp_ (mg/g)	R^2^
As(V)	12	10	1	0.004	3.25	3.08	0.911
Cu(II)	12	10	1	0.028	6.39	6.25	0.969
P(V)	12	10	1	0.013	1.46	1.32	0.979
