# Supplementary material for: Successive extraction of As(V), Cu(II) and P(V) ions from water using spent coffee powder as renewable bioadsorbents
Source: Sci Rep. 2017 Feb 21;7:42881. doi: 10.1038/srep42881 (PMC5318912; doi:10.1038/srep42881)
Supplement: Supplementary Information [file srep42881-s1.doc]

**Supplementary Information**

**Successive extraction of As(V), Cu(II) and P(V) ions from water using spent coffee powder as renewable bioadsorbents**

Linlin Hao^a,b^, Peng Wang^b^, Suresh Valiyaveettil^a*^

^a^Department of chemistry, National University of Singapore,

3 Science Drive 3, Singapore 117543.

^b^State Key Laboratory of Urban Water Resource and Environment

School ofMunicipal and Environmental Engineering, Harbin Institute of Technology,

Harbin, P. R. China 150090

Table S1. Comparison of the Langmuir capacity of different adsorbents for arsenic, copper and phosphate adsorption.

| Adsorbents | Adsorption capacity (mg/g) | | | |
| --- | --- | --- | --- | --- |
|  | As(III) | As(V) | Cu(II) | P(v) |
| Fe2O3 nanomaterial | 1.25^1^ | -- |  |  |
| Fe3O4 coated wheat straw | 3.9^1^ | 8.1^1^ |  |  |
| Iron modified polymer | 15.45^2^ | 7^2^ |  |  |
| Perlite/γ-Fe2O3 composite | -- | 4.64^3^ |  |  |
| Biochar/γ-Fe2O3 composite | -- | 3.15^4^ |  |  |
| ZVI nanoparticles modified starch | 12.2^5^ | 14^5^ |  |  |
| Coffee-PEI-Fe | -- | 83.3^6^ |  |  |
| Carboxyl-modified jute |  |  | 44^7^ |  |
| Wheatstraw based biochar |  |  | 33.3^8^ |  |
| Chitosan beads |  |  | 60.6^9^ |  |
| Surface-oxidized cellulose nanowhiskers |  |  | 20.7^10^ |  |
| Coffee-PEI-Fe |  |  | 200.1^6^ |  |
| Wheat residue based anion exchangers |  |  |  | 68^11^ |
| Al-intercalated acid activated bentonite beads |  |  |  | 23.6^12^ |
| Fe_3_O_4_/MgAl-NO_3_ layered double hydroxide |  |  |  | 34^13^ |
| Coffee-PEI-Fe |  |  |  | 50.2^6^ |

1. Tian, Y., et al., J. Hazard. Mater., 193 (2011) 10–16.
2. Daniel Ociński, et al., Reactive & Functional Polymers 83 (2014) 24-32.
3. Dong Nguyen, T., et al.,. Separation and Purification Technology 82 (2011) 93-101.
4. Ming Zhang, et al., Bioresource Technology 130 (2013) 457-462.
5. Mohammad Mosaferi, et al.[,](http://www.ijehse.com/content/12/1/74/#ins5) Journal of Environmental Health Science and Engineering, 12 (2014) 69-74.
6. This study
7. Du Zhaolin, et al., Bioresource Technology 201 (2016) 41-49.
8. A. Bogusz et al., Bioresource Technology 196 (2015) 540–549.
9. I. Sargın et al., Bioresource Technology 177 (2015) 1–7.
10. Hazren A. H. et al., Industrial Crops and Products 93 (2016) 108–120.
11. X. Xu et al., Carbohydrate Polymers 82 (2010) 1212–1218.
12. Radheshyam R. P. et al., Science of the Total Environment 572 (2016) 1222–1230.
13. Paulmanickam K. et al., Journal of Environmental Chemical Engineering 4 (2016) 984–991.
